# Are Protected Areas Required to Maintain Functional Diversity in Human-Modified Landscapes?

**DOI:** 10.1371/journal.pone.0123952

**Published:** 2015-05-06

**Authors:** H. Eden W. Cottee-Jones, Thomas J. Matthews, Tom P. Bregman, Maan Barua, Jatin Tamuly, Robert J. Whittaker

**Affiliations:** 1 Conservation Biogeography and Macroecology Group, School of Geography and the Environment, Oxford University Centre for the Environment, University of Oxford, Oxford, United Kingdom; 2 Azorean Biodiversity Group (ABG, CITA-A) and Portuguese Platform for Enhancing Ecological Research and Sustainability (PEERS), Depto de Ciências Agrárias, Univ. of the Azores, Rua Capitão João d´Ávila, Pico da Urze, PT-9700-042, Angra do Heroísmo, Portugal; 3 Edward Grey Institute, Department of Zoology, University of Oxford, Oxford, United Kingdom; 4 Wild Grass EcoLodge, Kaziranga, Assam, India; 5 Centre for Macroecology, Evolution and Climate, Department of Biology, University of Copenhagen, Copenhagen, Denmark; Chinese Academy of Forestry, CHINA

## Abstract

The conversion of forest to agriculture across the world’s tropics, and the limited space for protected areas, has increased the need to identify effective conservation strategies in human-modified landscapes. Isolated trees are believed to conserve elements of ecological structure, providing micro-sites for conservation in matrix landscapes, and facilitating seed dispersal and forest restoration. Here we investigate the role of isolated *Ficus* trees, which are of critical importance to tropical forest ecosystems, in conserving frugivore composition and function in a human-modified landscape in Assam, India. We surveyed the frugivorous birds feeding at 122 isolated *Ficus* trees, 33 fruit trees, and 31 other large trees across a range of 32 km from the nearest intact forest. We found that *Ficus* trees attracted richer and more abundant assemblages of frugivores than the other tree categories. However, incidence function estimates revealed that forest specialist species decreased dramatically within the first kilometre of the forest edge. Despite this, species richness and functional diversity remained consistent across the human-modified landscape, as habitat generalists replaced forest-dependent frugivores, and accounted for most of the ecological function found in *Ficus* trees near the forest edge. We recommend that isolated *Ficus* trees are awarded greater conservation status, and suggest that their conservation can support ecologically functional networks of frugivorous bird communities.

## Introduction

Agricultural conversion is a major driver of tropical forest destruction worldwide [1], to the extent that tropical agriculture now accounts for 13.3 m ha, an increase of 3% over the last decade [2]. With limited scope for the addition of new protected areas in many tropical regions, one of the major challenges in tropical conservation is devising strategies that can effectively conserve biodiversity in human-modified production landscapes [3].

Several studies have documented species loss and compositional shifts following the conversion of forest to agriculture [4], [5]. For birds, insectivorous species are often lost from human-modified landscapes [6], while forest-dependent species and large-bodied frugivores, which are particularly vulnerable to hunting [7], rarely venture beyond the forest edge [8]. The functional implications of these changes are, however, less clear [9]. Critical ecosystem functions, such as pollination and seed dispersal, may still be maintained by a depauperate assemblage of species [10]. Indeed in some human-modified landscapes, the frugivore–fruit tree relationship seems to be sufficiently intact to conserve seed dispersal mechanisms through networks of isolated trees [11]–[13]. Furthermore, isolated trees are argued to provide a range of other ecological benefits, including nesting and roosting sites, enhanced soil fertility, and sites for forest restoration, making them a potentially important biodiversity conservation agent in these landscapes [14].

In intact tropical forests, *Ficus* (Moraceae) trees are consistently identified as critically important ecological actors [15]–[17]. Their large crop size, aseasonal fruiting pattern, and nutritional composition make them a key resource for frugivores around the world’s tropics [18], [19]. Although usually studied in forest ecosystems, networks of isolated *Ficus* trees persist in many human-modified landscapes [12], [13], [20]. However, little work has been done to assess the status of the *Ficus*–frugivore relationship beyond the forest edge. Key questions, such as whether frugivores commute to fruiting trees from forest habitats, or independently reside in human-modified landscapes, remain unanswered [3]. Furthermore, few studies have explicitly considered agricultural habitats as conduits for the movement of seeds and frugivores, especially at the landscape scale [6], [21]. The lack of research on *Ficus*–frugivore interactions in human-modified landscapes is particularly concerning as the area required to support populations of the more sparsely distributed *Ficus* species over the long-term are likely to exceed the size of all but the largest protected areas [22]. Indeed the *Ficus*–frugivore relationship may only be sustainable, both within and beyond protected areas, through effective conservation in human-modified landscapes.

From a functional perspective, if the *Ficus*–frugivore relationship was dependent on protected areas, functional diversity would be expected to decline monotonically in relation to species loss as environmental conditions become increasingly unfavourable [23]. However, if frugivore assemblages utilizing *Ficus* trees are composed of random subsets of those species that occur across the landscape, then their functional diversity will be randomly distributed. On the other hand, if environmental filtering influences assemblage composition, we can expect a non-random distribution of functional diversity. Furthermore, the relationship between the decline in functional diversity and the decline in species richness should reveal the structure of species turnover. If species richness declines at a faster rate than functional diversity, it implies that functionally redundant species are lost first (functionally redundant species being those with traits shared with other species in the assemblage, [23]). If species richness declines at the same rate as functional diversity, the assemblages may be subject to random turnover, where no traits are particularly vulnerable to structural loss. Finally, where species richness declines at a slower rate than functional diversity, functionally unique species are lost first, indicating that rare traits are more vulnerable to loss through structured turnover, as hypothesized above.

To test the capacity of isolated *Ficus* trees to conserve the *Ficus*–frugivore relationship in human-modified landscapes, we examined three hypotheses. First, given the importance of *Ficus* trees to birds in intact forests, we hypothesise that isolated *Ficus* trees will have a higher abundance and diversity of frugivorous birds than other types of isolated tree. Second, in determining the composition of assemblages at isolated *Ficus* trees, we posit that the distance of a *Ficus* tree from the nearest forest will have the strongest influence on frugivore assemblages. Finally, we hypothesise that functionally unique frugivore species are more vulnerable to extirpation in human-modified landscapes, and so will be lost first from isolated *Ficus* trees.

## Materials and Methods

### Study area

Our study was conducted in the Golaghat District of Assam, North-east India. This region’s original moist subtropical forest was largely cleared following the local commercialisation of tea production around 1840 [24]. The study area of ≈250 km^2^ extends between Kaziranga National Park (N26 34.394 E93 15.433), the town of Golaghat (N26 27.819 E93 54.978), and Jorhat (N26 46.198 E94 12.678). Aside from Kaziranga National Park, additional protected areas in the study area were Panbari Forest Reserve (N26 37.025 E93 30.963), at the foot of the Karbi Hills, and Nambor Wildlife Sanctuary (N26 28.769 E93 48.687), south of Golaghat.

Humans have heavily modified the landscape across the study area, so that it now forms an agricultural mosaic with a heterogeneous assortment of small-holder rice cultivation, tea estates, and village home gardens. The region has a population density of 302 people / square kilometre [25]. The elevation of the study area ranges between 30 and 100 m above sea level, and the mean annual rainfall for the region is 1,500–2,500 mm, most of which falls in the May to August monsoon [24]. The annual temperature range varies from an average absolute minimum of 5°C to an average absolute maximum of 35°C [24].

### *Ficus* data collection

**Fig 1:**
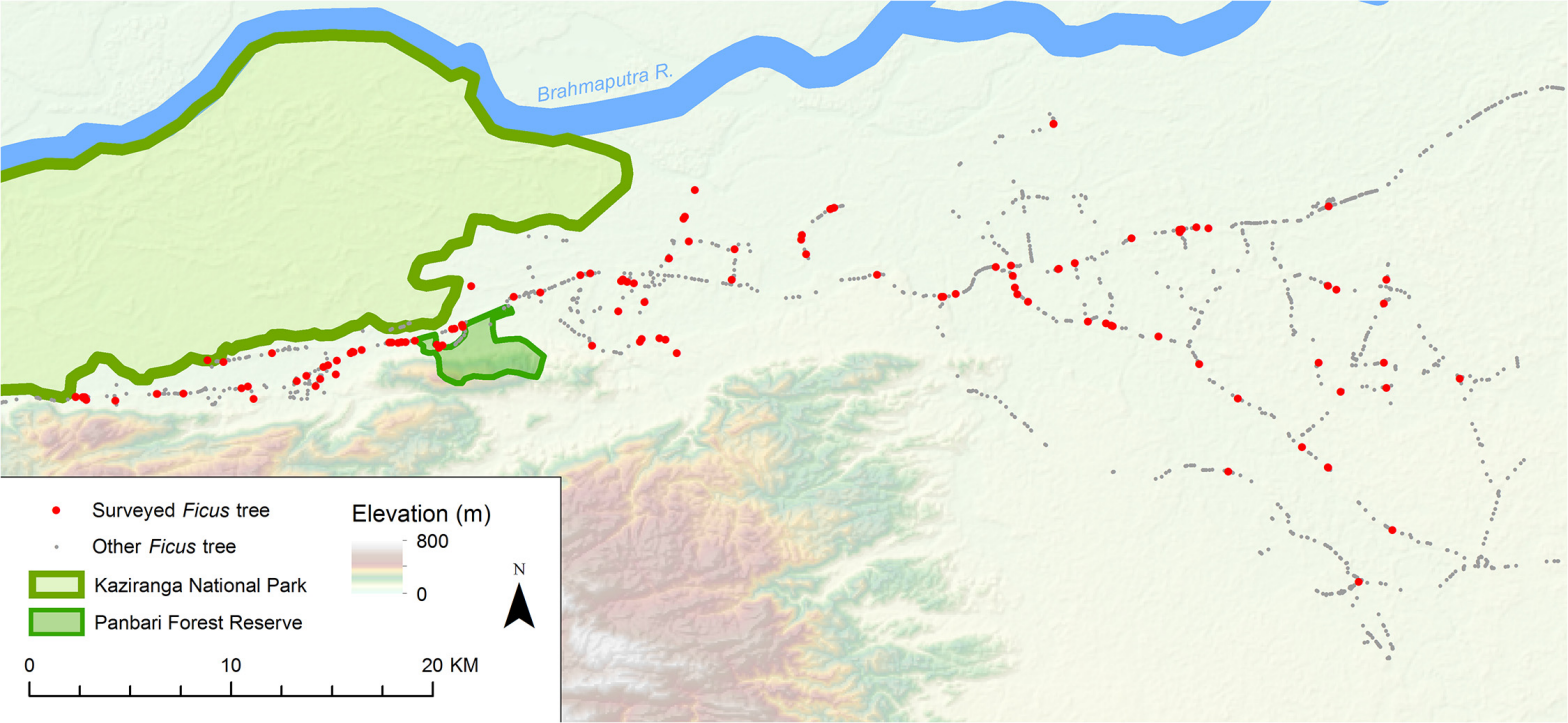
Locations of the 1,857 *Ficus* trees marked in the study area. Red circles denote surveyed trees, grey circles denote trees that were not surveyed. The linear pattern at this scale indicates the association between roads and the distribution of *Ficus* trees. Kaziranga National Park and Panbari Forest Reserve contain the only relatively intact forests in the study area.

Field data were collected between September 2009 and June 2013. We first carried out a thorough search of the area by car and foot, marking all mature *Ficus* trees with a GPSmap 62s device, typically accurate to ≤ 5 m in the open habitats the *Ficus* trees were situated in. In total, 1,857 *Ficus* trees were located (Fig 1). The most common *Ficus* species encountered were *F*. *religiosa* and *F*. *benghalensis*, followed by *F*. *rumphii*, *F*. *microcarpa*, *F*. *racemosa*, *F*. *benjamina*, *F*. *elastica*, and *F*. *assamica*. Due to the different life history of *F*. *assamica*, it was excluded from the mapping exercise. The mapped trees were regularly checked to monitor fruit ripeness. When a tree produced a ripe crop, we measured its diameter at breast height (DBH), height, and canopy diameter along two axes. Canopy area was later calculated using the formula for an ellipse. The average height of measured *Ficus* trees was 26.58±0.72 m, (mean±SE, from a sample of 122 surveyed *Ficus* trees), with a mean DBH of 1.42±0.06 m, and canopy area of 474.02±29.68 m^2^. To produce a single measure for overall tree size, a Principal Components Analysis (PCA) with Kaiser stopping criterion extraction (eigenvalues >1) was conducted using DBH, height, and canopy area in IBM SPSS Statistics 22 [26]. As the input variables were correlated with each other we used an oblique rotation method (“Direct Oblimin” in SPSS). The observed intensity of human land-use within a 100 m radius of the tree was recorded using a three-point scale (where 0 is very little human land use; 1 is some human land use, such as a village home garden or livestock grazing area; and 2 is intense human land use, in cases where a road, house, or paddy field were present). The size of fruit (for figs properly termed syconia) produced by the tree was categorised as either large (mean diameter > 150 mm) or small (< 150 mm), by measuring three recently fallen fruit. We measured the distance to the nearest protected area with intact forest by overlaying the *Ficus* GPS markers on Landsat 8 satellite images of the region in ArcGIS 10.2.1 [27], and then digitised the protected area borders through an on-screen visual interpretation. The distance of each *Ficus* tree to the nearest protected area was then measured in kilometres using ArcGIS. In all cases, protected areas held the only high-quality forest habitat left in the study landscape. In addition to protected areas, small (≤1 ha), low-quality wooded areas were located through consultation with local landholders and marked with a GPS device. In cases where *Ficus* trees were closer to small, low-quality wooded areas than protected areas, additional measurements were made following the above procedure to estimate the distance to the nearest wooded area of any quality.

### Frugivore data collection

Fruiting *Ficus* trees were observed to have a large ripe crop for 3–7 days. During this period we would conduct one frugivore survey per tree. Single-tree surveys are increasingly used in ecological studies (e.g. [11], [28]), and can provide detailed results for the target taxa. Frugivore surveys commenced at first light (which ranged from 04:20–05:20 depending on the season) and continued for three hours. Previous studies of *Ficus* trees in the study area had demonstrated that few new bird species were added by repeat surveys, and that three hours was the optimum length of time to record frugivore assemblages before activity decreased as the sun rose [29]. Surveys were only conducted in fair weather conditions. If the weather deteriorated during the survey period, the survey was abandoned and attempted again on the following day. During each survey, an observer would watch the tree from a concealed position with a good view, typically about 20 m from the trunk. Each individual bird that landed in the tree was recorded, including the time, direction, and distance of arrival. Birds that made repeated visits to and from the tree were recorded with an asterisk to avoid double counting. In situations where too many birds were arriving and leaving the tree to accurately count, the highest number of birds recorded in any one instance was used for analysis.

The same protocols were used to survey control groups of non-*Ficus* fruiting trees (“fruit trees”) and other large non-fruiting trees (“large trees”), to test if *Ficus* trees were more attractive to frugivores than other tree types. The trees selected for these control surveys were also commonly encountered species in the human-modified landscape. Although the signs of ripeness vary between species, we only surveyed fruit trees when they produced a ripe crop, which could be verified by observing birds feeding on the fruit the afternoon prior to the frugivore survey. Other large trees were selected on the basis of being the largest trees in the area, as judged by height. Their classifications and attributes are provided in Table 1.

**Table 1:** Characteristics of the isolated *Ficus* trees and the two control tree categories included in the study.

Characteristic	*Ficus*	Fruit	Large
Total no. of individuals surveyed	122	33	31
Total no. of species surveyed	6	12	15
DBH (m)	1.42±0.06 ^a^	0.45±0.02 ^b^	0.61±0.05 ^c^
Height (m)	26.58±0.72 ^a^	18.86±1.03 ^b^	20.91±0.89 ^b^
Canopy area (m^2^)	474.02±29.68 ^a^	74.01±7.16 ^b^	130.11±21.43 ^c^
Five most surveyed species (in order of decreasing abundance)	*F*. *religiosa*, *F*. *benghalensis*, *F*. *rumphii*, *F*. *microcarpa*, *F*. *benjamina*	*Artocarpus heterophyllus*, *Tectona grandis*, *Artocarpus lakoocha*, *Syzgium cumini*, *Toona ciliata*	*Syzgium cumini* (non-fruiting), *Albizia lucidor*, *Albizia procera*, *Mangifera indica* (non-fruiting), *Neolamarckia cadamba*

DBH is diameter at breast height. Values for DBH, height, and canopy area are mean ± standard error. Different superscript letters denote significantly different means at *p*<0.05 following ANOVA and Games–Howell post hoc tests. The five most surveyed species are listed in order of decreasing number of surveys.

### Frugivore classifications

**Fig 2:**
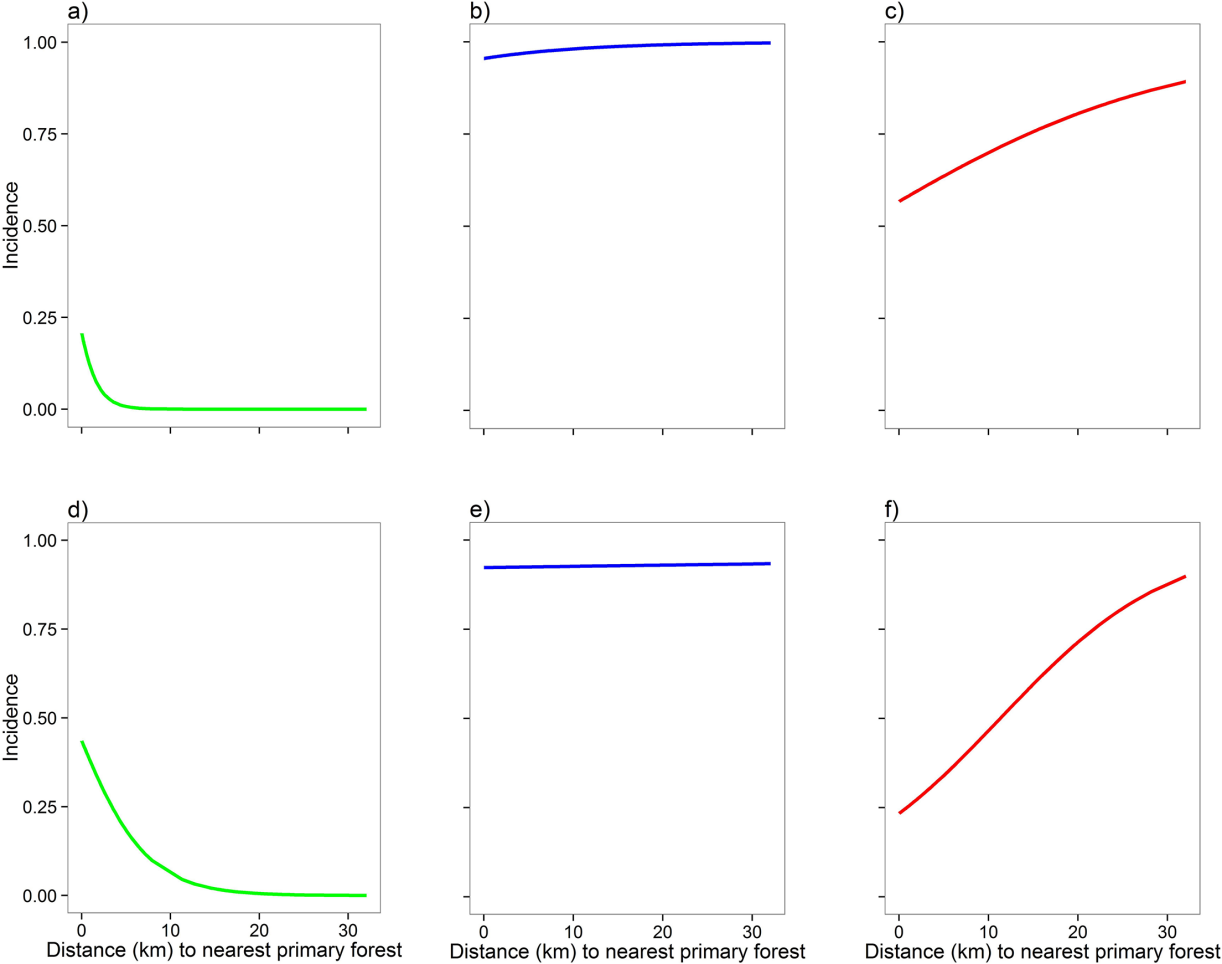
Incidence functions for six frugivore species. Species are a) Great Pied Hornbill *Buceros bicornis*; b) Red-vented Bulbul *Pycnonotus cafer*; c) Great Myna *Acridotheres grandis* d) Blue-eared Barbet *Megalaima australis*; e) Coppersmith Barbet *Megalaima haemacephala*; f) Black-hooded Oriole *Oriolus xanthornus*. Green curves represent forest-dependent species, blue represent habitat-generalists, and red represent matrix-specialists. The curves are the predicted probability of occurrence, generated through a logistic regression model, with distance from the nearest primary forest as the predictor variable for each species’ presence/absence data at 122 isolated *Ficus* trees. The incidence function curves of all 33 frugivore species are displayed in S1 Fig. The figures were constructed using the R package “GGplot2” [33].

Immediately after the frugivore survey, the number of birds of each species recorded visiting the tree would be totalled. Nomenclature followed [30] for the most recent detailed review of Indian bird taxonomy. Each species was classified into primary and secondary dietary guilds (frugivore, nectivore, insectivore, granivore, or carnivore) following [31], [32]. We also used these sources to classify each species’ habitat preference as forest-dependent, habitat-generalist, or matrix-specialist. To cross-check the local validity of these classifications, binary logistic regression models were run for each species (excluding singletons and doubletons), with presence or absence in a *Ficus* tree as the response variable, and the distance from protected area as the predictor variable. The resulting predicted probabilities of occurrence were then used to plot incidence functions against distance for each species (Fig 2 and see S1 Fig) using the R package “GGplot2” [33]. Sharp downward curves were indicative of forest-dependent species, flat curves (no change of more than 10% over 30 km) denoted generalists, and rising curves were considered characteristic of matrix-specialists.

### Ecomorphological data collection

In order to obtain ecomorphological trait data, specimens of each species recorded in the surveys were measured following Edward Grey Institute protocols at the British Natural History Museum’s ornithological collections. We defined a “trait” as a measurable aspect of an organism that in part determines its interaction with the environment [23]. Here we were interested in the foraging and dispersal capacity of frugivorous birds, so we measured traits associated with locomotive behaviour, dispersal ability, gape size, bill structure, and body size [34], [35]. Specifically, we measured four specimens of the correct local subspecies for each species recorded. In almost all cases we were able to measure specimens collected within 150 km of the study area. Two adult males and two adult females of each species were measured with 150 mm outside diameter dial callipers (accurate to 0.1 mm), wing rulers, and tail rulers (accurate to 0.5 mm) from Porzana Ltd. The measurements taken were: culmen length (from the base of the skull to the tip of the bill), bill length from nares (from the anterior of the nares to the tip of the bill), bill width (the width of the bill at the anterior of the nares), bill depth (the depth of the bill at the anterior of the nares), gape width, tarsus length (the length from the inner bend of the tibiotarsal articulation to the base of the toes, where the scalation pattern changes), wing chord (from the bend in the wing to the unflattened longest primary), Kipp’s distance (the distance from the longest primary to the first secondary), and tail length (to the tip of the longest retrix). Bill measurements excluded ornamentation, which meant the casques of *Buceros bicornis* and *Anthracoceros albirostris* were not included in bill width or depth measurements. As weight data for birds are often variable [36], we preferred to measure body size through a PCA using SPSS [26]. We first conducted a PCA of tail length, wing chord, and tarsus length, using oblique rotation with Kaiser stopping criterion extraction (eigenvalues >1), which produced two components. The second of these was used as an index for locomotive ability, while the first related to body size. We then ran a PCA with bill depth, width, and length from nares using the same procedure, obtaining two components: the first related to size again, while the second was used as an index of bill shape. The first principal components of the two analyses were then used in isolation for a third PCA, which produced an index for overall body size. To create an index for dispersal ability that standardizes for bird size, we calculated the hand-wing index [35], which is a surrogate for flight performance, migratory behaviour, and natal dispersion in birds. Only species observed eating *Ficus* fruit were retained for further analysis.

### Functional dispersion

We calculated the functional diversity of the frugivores recorded at each *Ficus* tree to identify trends in the provision of ecological services, and to test for the existence of environmental filtering in frugivore assemblage composition of isolated *Ficus*. By using trait information to define a species’ ecological role within a community, a single continuous metric can be produced, which permits an assessment of functional redundancy and structured turnover in assemblages across environmental gradients [9]. We follow the definition of functional diversity as the distribution of functional traits within multidimensional niche space [37], and used Laliberté and Legendre’s functional dispersion (FDis) index to measure functional diversity in our dataset [38]. This represents the spread of the species in quantitative trait space by calculating a multidimensional index of the mean distance of an individual species to the centroid of all species in the community [38]. A major advantage of FDis over other measures, such as FRic, FEve, and FDiv [39] is that it can be calculated for communities composed of only two species, rather than a minimum of three. It is also independent of species richness, and can be weighted by abundance, both of which were important considerations for our study [38]. We calculated functional dispersion for each of our *Ficus* trees using the R package “FD” [40], [41].

In the absence of strong ecological reasons to weight our data, we used an unweighted trait matrix [37]. As our trait data were measured on a continuous scale, rather than classified into nominal groups, a species–species uncorrected distance matrix was computed. A principal co-ordinates analysis (PCoA) was performed after the distance matrix was corrected for negative eigenvalues [40] to avoid introducing a bias to the functional dispersion estimates. These corrected PCoA axes were used to calculate the functional dispersion scores for our *Ficus* trees in SPSS [26], [40].

### Statistical analysis

To assess the importance of *Ficus* trees to frugivores against the two control groups (other fruit trees and large trees), we compared the estimated richness, Shannon Index, observed species richness, abundance, and functional dispersion parameters across the three tree categories. Richness was estimated using the Chao 1 bias corrected estimator, which uses the number of singletons and doubletons to estimate the number of undetected species. The Shannon Index scores were derived from Chao and Shen’s [42] revised algorithm. Both the richness estimator and Shannon Index were computed in SPADE [43]. An analysis of variance (ANOVA) test was used to examine differences between observed species richness and abundance over the three tree categories. The data were log transformed for normality, and tested for homogeneity of variance using Levene’s test. In cases where equal variances could not be assumed, Welch’s F-ratio was used to identify the main effects at the *p* = 0.05 level of significance. A Games–Howell post hoc test, which is robust to unequal sample sizes, was used to identify significant differences between groups [44]. Functional dispersion (FDis) did not conform to normality even after transformation in the fruit or large tree categories, so we used a Kruskal–Wallis test with Mann–Whitney follow-up procedures and a Bonferroni correction of *p*<0.0167. We also examined species richness, abundance, and FDis across the three tree types, while controlling for the canopy area of each tree (see S1 Appendix). All ANVOAs and non-parametric equivalents were conducted in SPSS 22.0 [26].

We estimated Morisita’s similarity index to assess the similarity between the frugivore assemblages recorded at *Ficus* trees and the two control groups in SPADE. This index estimates the similarity of multiple communities from abundance data, taking into account unseen shared species. It performs better than traditional pair-wise similarity indices as it considers information shared by more than two communities, especially in cases where there are numerous rare species [45]. We randomly selected 31 surveys from each tree category (all surveyed during the same season) and summed the abundance of each bird species to produce equal sample sizes.

Although we expected some structural relationships in our data, as trees that were close to the primary forest block were also close to each other, we tested the degree of spatial autocorrelation using Moran’s *I* coefficient test with arbitrary distance classes and a Bonferroni correction in R (using package “ape” [46]). We ran the test for frugivore abundance, richness, and functional dispersion in the 122 *Ficus* trees.

To identify the factors that influence frugivore assemblage composition in isolated *Ficus* trees, we used an information-theoretic approach to test the effect of distance from the nearest protected area, land-use intensity, tree size, fruit size, and season on frugivore richness, abundance, and functional dispersion. Distance to the nearest protected area was selected over distance to the nearest forest of any quality as it demonstrated a better fit with the response variables in initial analyses (S2 Appendix). Land-use intensity, fruit size, and season were categorical predictor variables. To determine “season”, the months *Ficus* trees were surveyed were divided into winter (November–March), early monsoon (April–July), and late monsoon (August–October), to reflect the passage of migrants observed in the study area during field data collection. A generalized linear model (GLM) with a log-link function and Poisson error distribution was run for richness and abundance data, while the functional dispersion analyses used an identity link function with Gaussian error [47]. Combinations of the five predictor variables and their second-order interaction terms were evaluated using a second-order criterion (AIC_c_) to select the best model notwithstanding the small ratio between the number of input variables and observations [47]. The model with the lowest AIC_c_ score was taken to signify the best performing model, although all models within <2 ΔAIC_c_ of the best performing model were considered to have similar support [47].

To investigate the relationship between frugivore abundance and distance from the nearest primary forest we ran a boundary analysis with a randomly distributed null model. This test can indicate whether a particular quadrant in ecological space is significantly under- or over-populated than expected by chance, taking into account both the number of data points that fall within a predetermined quadrant, and the distances of each of those points to the boundary of that quadrant. We ran 1,000 iterations of an asymmetrically distributed left triangle in the upper right quadrant in the EcoSim software package [48].

We also tested the significance of distance in frugivore abundance patterns by conducting a quantile regression [49]. This technique fits regression curves to different parts of the response variable’s distribution, and is particularly useful in situations with heterogeneous variance [50]. We plotted curves for seven quantiles (0.05, 0.10, 0.25, 0.50, 0.75, 0.90, and 0.95) using the R package “quantreg” [49]. We identified significant differences by plotting the mean slope against those for each quantile, where quantiles with 95% confidence intervals that did not overlap with the mean slope were taken to be significantly different.

To investigate the response of individual frugivore species to the four environmental predictors (distance from nearest primary forest, tree size, fruit size, and land-use intensity) we conducted a Canonical Correspondence Analysis in R using package “vegan” [51]). We did not include “season” as this cannot be affected by conservation actions. By performing a weighted linear regression on the constraining predictor variables, this method is useful for testing the *a priori* hypotheses of important constraints generated through extensive field observations during data collection.

To assess whether trees supported higher or lower levels of functional diversity than would be expected by chance, we compared observed functional dispersion patterns with those of null model communities generated using the sum-of-squares reduction method (“quasi-swap”, [52]). With species richness held constant for each tree, and frugivore incidence held constant for each species, the model randomly simulated null communities from the species pool (the total number of frugivores recorded in the study). We ran 10,000 simulations and tested whether the observed functional dispersion of each *Ficus* tree was significantly higher or lower than the null distribution at *p* = 0.05, using package “vegan” in R [41], [51]. We used a paired two-tailed Wilcoxon signed-rank test to identify significant differences between the observed and expected FDis scores.

### Ethics Statement

This research was conducted with ethical approval from the University of Oxford (Departmental CUREC reference number: SOGE C1A-99). Frugivore surveys were conducted with permission from local landowners where necessary, and permission to conduct this field work in India was granted by the High Commission of India, London, under visa number 4246496. Field studies did not involve endangered or protected species.

## Results

In 122 surveys of fruiting *Ficus* trees (totalling 366 hours of observation) we recorded 98 bird species, 33 of which were frugivores that were observed eating *Ficus* fruit. Three species, the Oriental White-eye (*Zosterops palpebrosus*), Yellow-vented Flowerpecker (*Dicaeum chrysorrheum*), and Scarlet-backed Flowerpecker (*Dicaeum cruentatum*) are described as being at least partially frugivorous [32], but were not observed eating figs during our surveys. In total, 30,084 (mean = 246.59) individual frugivores were recorded visiting *Ficus* trees during the surveys. In addition, 33 fruit trees and 31 large trees were surveyed, which produced 460 (mean = 13.94) and 224 (mean = 7.23) individual frugivore records, respectively.

The incidence function results demonstrated that the highest proportion of species recorded in *Ficus* trees were forest-dependent frugivores (15/33, 45.46%), followed by matrix-specialists (10/33, 30.30%), and habitat-generalists (8/33, 24.24%) (S1 Fig illustrates the incidence functions of all 33 species).

The comparison of *Ficus* trees versus other fruit trees and large trees indicated that *Ficus* trees have significantly richer frugivore assemblages, with, on average, a higher abundance of frugivores (Table 2). Specifically, the number of bird species differed between the groups (F_2,183_ = 200.05, *p*<0.001, ω^2^ = 0.47), with significant differences between *Ficus* trees and the other two categories (*p*<0.001), and no difference between other fruit trees and large trees (*p* = 0.43). The mean abundance of all birds in the three groups was significantly different (Welch’s F_2,50_ = 219.59, *p*<0.001, ω^2^ = 0.87). The Games–Howell test revealed that *Ficus* trees had higher frugivore abundance than the other groups (*p*<0.001 in both cases), while other fruit trees and large trees were not significantly different (*p* = 0.1). The same pattern was found for functional diversity, with *Ficus* trees having significantly higher functional dispersion than fruit or large trees (H_2_ = 43.29, *p*<0.001; *Ficus* vs fruit: U = 898, *p*<0.001, mean *Ficus* rank = 87.14, mean fruit rank = 44.21; *Ficus* vs large: U = 724, *p*<0.001, mean *Ficus* rank = 86.57, mean fruit rank = 39.35), again with no statistical difference between the latter two categories (U = 433, *p* = 0.28).

**Table 2:** Differences between species richness, abundance, and functional diversity parameters across the three tree categories.

Parameter	*Ficus*	Fruit	Large
Estimated richness	26.00±4.60 ^a^	17.00±0.30 ^b^	18.20±0.50 ^b^
Shannon Index	2.07±0.02 ^a^	2.22±0.04 ^b^	2.27±0.07 ^c^
Observed richness	13.02±0.27 ^a^	3.97±0.58 ^b^	2.97±0.56 ^b^
Abundance	246.59±18.39 ^a^	13.94±3.27 ^b^	7.23±1.85 ^b^
FDis	0.88±0.02 ^a^	0.64±0.10 ^b^	0.46±0.10 ^b^

Values are means ± standard error. Different superscript letters denote significantly different means. Estimated richness is the Chao 1 bias corrected estimator, which uses the number of singletons and doubletons to estimate the number of undetected species [43]. The Shannon Index scores are derived from the Chao and Shen [42] revised algorithm. Observed richness is the average number of frugivorous bird species recorded in the surveys of each tree category. Abundance is the average number of individual frugivores recorded in the surveys of each tree category. FDis is a multidimensional index of the mean distance of an individual species to the centroid of all species in the community [38]. Estimated richness and Shannon scores were considered to be significantly different where 95% confidence intervals did not overlap. For species richness and abundance, different superscript letters denote significantly different means at *p*<0.05 for species richness and abundance using ANOVA with a Games–Howell post hoc test. For FDis, differences were tested using a Kruskal–Wallis test with Mann–Whitney follow-up procedures, using a Bonferroni correction of *p*<0.0167.

The Morisita similarity estimates of the abundance data indicate a moderately high level of similarity between the frugivore communities recorded in fruit and large trees. *Ficus* trees had very low estimated similarity with members of the other two groups (Table 3).

**Table 3:** Morisita similarity matrix of multiple communities, estimated from the abundance of frugivores recorded in three tree types (n = 31 surveys for each group).

	*Ficus* trees	Fruit trees	Large trees
***Ficus* trees**	1.0	0.32±0.01	0.31±0.02
**Fruit trees**		1.0	0.94±0.02
**Large trees**			1.0

Values are Morisita similarity estimate ± standard error, with 200 bootstrap replications. Values closer to 1.0 indicate higher community similarity. Average pairwise comparison = 0.52.

The Moran’s *I* results indicated that there was no spatial autocorrelation at any scale for frugivore richness, with no consistent trend in the *I* coefficients, and no *p* values <0.05. Frugivore abundance and functional dispersion displayed similar patterns to each other, with significant clustering at the largest spatial scales, before a non-significant ‘trend’ of slight dispersion at medium and small spatial scales.

In testing the determinants of frugivore richness at isolated *Ficus* trees, the best performing GLM included distance, tree size, and season as predictor variables (Table 4). Specifically, frugivore richness increased as tree size increased, but decreased as the distance between the *Ficus* tree and the nearest protected area increased (Table 5). Significantly more species were present in *Ficus* trees in the early monsoon compared to winter. Frugivore abundance also fluctuated seasonally, with more frugivores present in the early and especially the late monsoon. Again, increasing distance caused a decrease in abundance, as did medium and high land-use intensities compared to low land-use intensity, and large fruit size compared to small. Increasing tree size also increased the number of frugivores visiting isolated *Ficus* trees. Reflecting the fluctuations in species richness and abundance with season, FDis varied seasonally, and also decreased as distance increased.

**Table 4:** Generalized Linear Model results with Akaike Information Criterion scores for finite samples for variables affecting three measures of frugivore assemblage at isolated *Ficus* trees in Assam, India.

Variable	Model	K	MML	AIC_c_	ΔAIC_c_	*w*_*i*_
**Richness**	**S, M, D**	**3**	**-299.51**	**609.54**	**0**	**0.46**
	S, M	2	-301.69	611.72	2.18	0.16
	D, M	2	-301.96	612.27	2.73	0.12
**Abundance**	**D, S, L, F, M**	**5**	**-5233.41**	**10482.10**	**0**	**1.00**
	D, S, L, F	4	-5415.19	10843.11	361.01	0.00
	D, L, M*S	3	-5425.34	10865.66	383.56	0.00
**FDis**	**D, M**	**2**	**53.70**	**-96.88**	**0**	**0.24**
	M	1	52.46	-96.59	0.29	0.20
	L, M	2	54.14	-95.55	1.33	0.12
	M, F	2	52.84	-95.15	1.73	0.10

“Richness” and “Abundance” were modeled using a log-link model with Poisson error distribution. “FDis” reports the AIC_c_ results for frugivore functional dispersion, using an identity link function. Data are derived from 122 frugivore surveys in Golaghat District, Assam, India. Model input abbreviations are L = land-use intensity; D = distance from nearest forest; S = tree size; F = fruit size; M = season. M*S is an interaction term between season and tree size. Other denotations: K = parameters in the model; MML = Maximum Log-likelihood; AIC_c_ = second-order Akaike Information Criterion score for finite samples; ΔAIC_c_ = the difference in AIC_c_ scores compared to the “best” performing model; *w*_*i*_ = Akaike weight, the normalized model likelihoods [47]. All models <2 ΔAIC_c_ are presented for each response variable, or if fewer than three models had a ΔAIC_c_ of <2, the three models with the most parsimonious fits are presented. In each case the “best” performing model is highlighted in bold font.

**Table 5:** Correlation coefficients for the parameters retained in the “best” performing models (see Table 4).

Variable	Parameter	B (estimate)±S.E.	Wald chi-square	*p*
**Richness**	Intercept	2.52±0.05	2250.54	<0.001
	Season (late monsoon)	0.01±0.08	0.01	0.94
	Season (early monsoon)	0.22±0.06	13.63	<0.001
	Season (winter)	-	-	-
	Tree size	0.06±0.02	5.03	<0.05
	Distance	-0.01±0.003	4.34	<0.05
**Abundance**	Intercept	5.77±0.01	198404.11	<0.001
	Fruit size (large)	-0.22±0.01	297.02	<0.001
	Fruit size (small)	-	-	-
	Distance	-0.03±0.001	962.31	<0.001
	Land-use intensity (high)	-0.10±0.01	55.33	<0.001
	Land-use intensity (medium)	-0.69±0.02	673.07	<0.001
	Land-use intensity (low)	-	-	-
	Season (late monsoon)	0.29±0.02	353.86	<0.001
	Season (early monsoon)	0.08±0.01	31.93	<0.001
	Season (winter)	-	-	-
	Tree size	0.24±0.01	2018.32	<0.001
**FDis**	Intercept	0.91±0.03	964.37	<0.001
	Season (late monsoon)	-0.11±0.04	6.76	<0.01
	Season (early monsoon)	0.04±0.03	1.65	0.20
	Season (winter)	-	-	-
	Distance	-0.003±0.002	2.50	0.11

“Richness” reports frugivore richness, “Abundance” reports frugivore abundance, and “FDis” reports frugivore functional dispersion from isolated *Ficus* trees in Assam, India.

**Fig 3:**
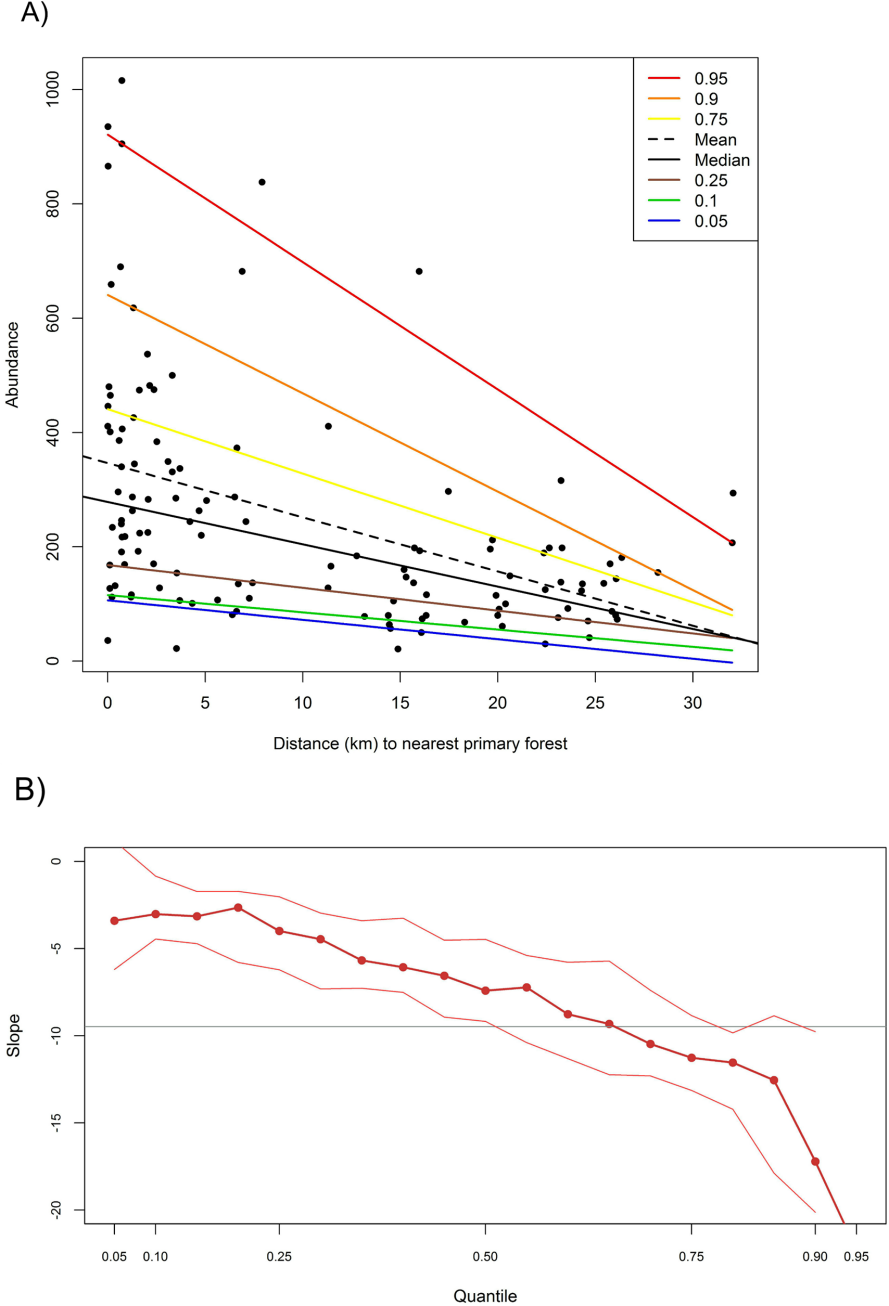
Quantile regression estimation of changes in frugivore abundance in isolated *Ficus* trees as a function of distance from the nearest primary forest in rural Assam, India. A) is a scatter plot of n = 122 isolated *Ficus* trees with 0.05, 0.10, 0.25, 0.50 (median), 0.75, 0.90, and 0.95 quantile and the least squares mean regression estimates. B) shows the sample estimates for the slope (thick red line) with thin red lines connecting the endpoints of the 95% confidence intervals. The grey line represents the mean slope. The figures were constructed using the R package “GGplot2” [33].

The further examination of changes in frugivore abundance with distance indicated that significantly fewer frugivores occurred in *Ficus* trees at long distances from source forest than would be expected by chance (boundary test: number of points, observed<expected, *p*<0.05; sum of squares, observed<expected, *p*<0.01). The quantile regression indicated that there was heterogeneous variance in frugivore abundance (Fig 3A). The lower and median quantiles (0.05, 0.10, 0.25, and 0.50) were significantly flatter than the mean slope (Fig 3B), indicating that some frugivores were present in similar numbers irrespective of distance. However, the higher quantiles (0.90, 0.95) were also significant, reflecting the marked decrease in occurrence of the highest frugivore abundances as distance increased. These two findings correspond with the incidence function results, where generalists were observed at all trees across all distances, while forest-dependent species (which in the case of the *Treron* pigeons are large flocking species), declined sharply with distance.

**Fig 4:**
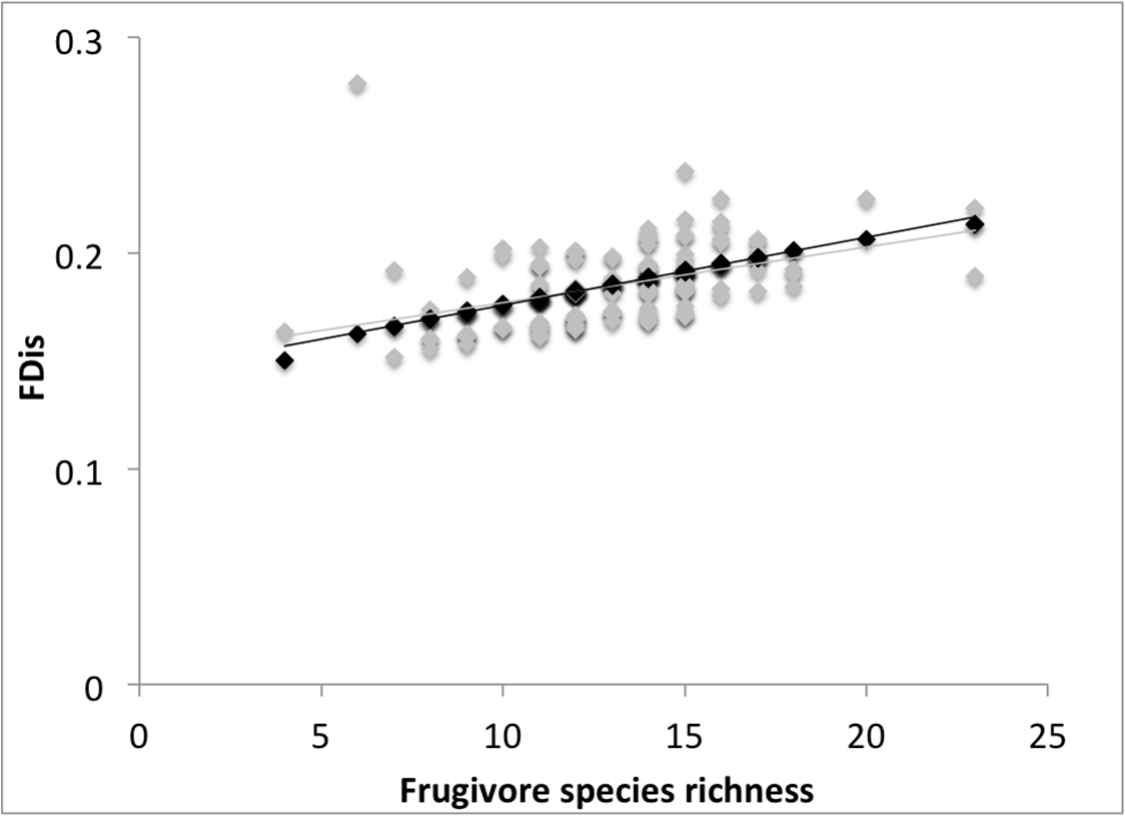
Scatter plot of observed (grey points) versus expected (black points) functional dispersion for frugivorous birds in isolated *Ficus* trees. The sample size was 122 isolated *Ficus* trees in a human-modified landscape in Assam, India. Trend lines are linear regressions for both observed FDis (grey line; *R* ^2^ = 0.16) and expected FDis (black line; *R* ^2^ = 0.99). Expected FDis scores are the mean of 10,000 iterations of a quasi-swap null model, where row and column totals were held constant.

The null model results suggested that isolated *Ficus* trees did not have higher or lower FDis than expected by chance (with species numbers held constant; Z = -1.12, *p* = 0.27). Only one of the 122 assemblages had values significantly different from random (0.82%), and this assemblage had a lower expected mean than observed FDis score. Furthermore, observed functional dispersion declined monotonically, and did not differ from the expected functional dispersion at low or high species richness (Fig 4), refuting the notion that functionally unique or functionally redundant species may have been lost first through structured turnover.

**Fig 5:**
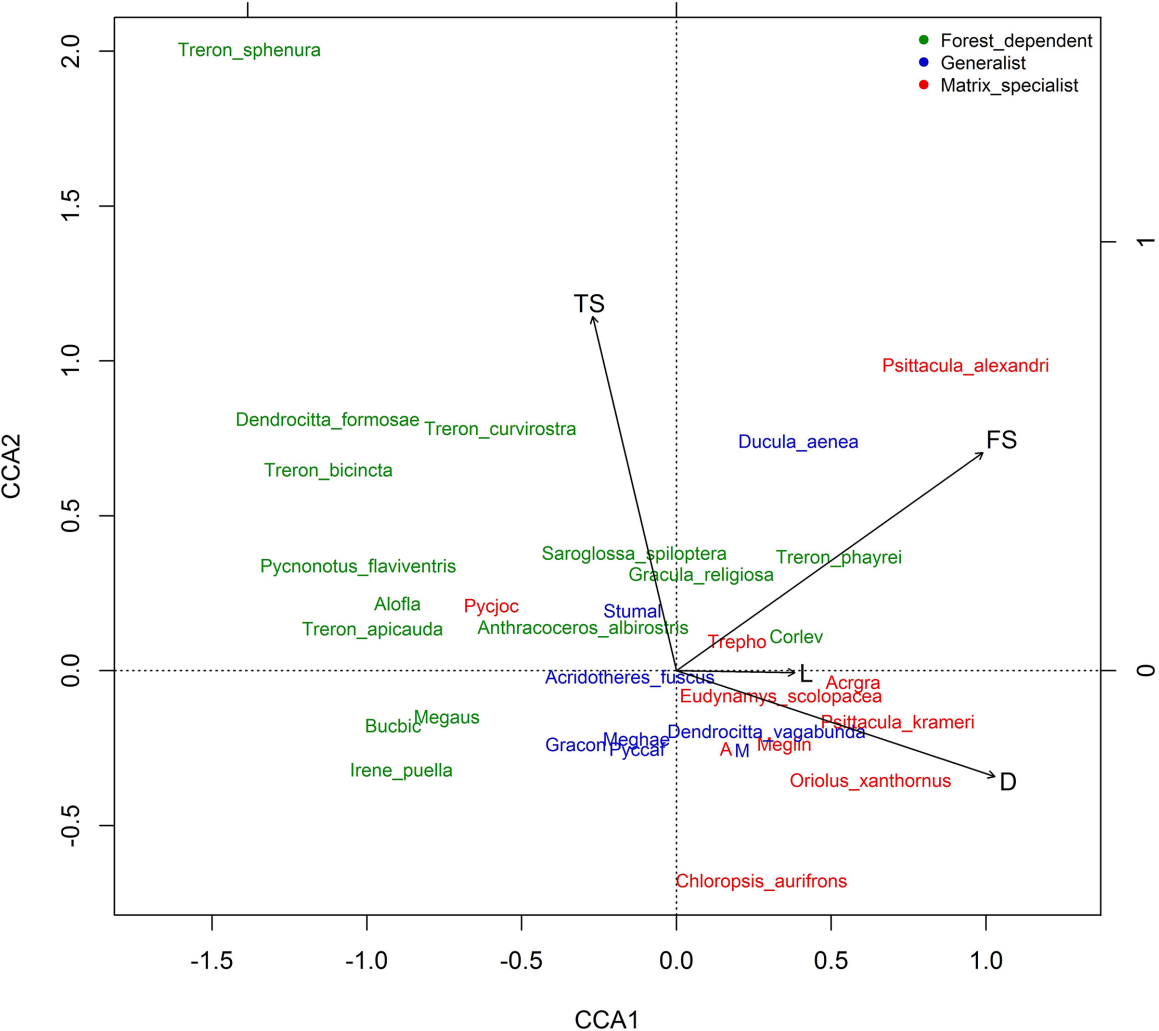
Canonical correspondence analysis showing the relationships between the abundance of individual frugivorous species and constrained environmental parameters. The direction of influence of the environmental parameters is indicated by the solid black lines, and annotations are: TS = tree size, FS = fruit size, L = land-use intensity, D = distance from nearest primary forest. Species names are colour coded according to classifications of habitat preference drawn from the literature. Common names are provided in S1 Fig. Some species names are abbreviated to aid interpretation. They are: Alofla = *Alophoixus flaveolus*, Pycjoc = *Pycnonotus jocosus*, Bucbic = *Buceros bicornis*, Megaus = *Megalaima australis*, Stumal = *Sturnia malabarica*, Gracon = *Gracupica contra*, Meghae = *Megalaima haemacephala*, Pyccar = *Pycnonotus cafer*, Trepho = *Treron phoenicoptera*, Corlev = *Corvus levaillantii*, A = *Acridotheres tristis*, M = *Megalaima asiatica*, Meglin = *Megalaima lineata*, Acrgra = *Acridotheres grandis*.

The Canonical Correspondence Analysis illustrated three important trends in species composition on isolated *Ficus* trees (Fig 5). First, it corroborated the incidence function analysis in highlighting the importance of distance in structuring the community. Forest-dependent species were negatively related to distance, with generalists showing no strong relationship, and matrix-specialists displaying a positive relationship. Tree size was also important, particularly for the *Treron* fruit doves, which form large flocks and so seem to prefer larger trees, which would theoretically provide a larger food resource. Interestingly, the largest species in the assemblage, the Great Indian Hornbill (*B*. *bicornis*), was not strongly associated with tree size. Few species associated with land-use intensity, although the Great Myna (*Acridotheres grandis*), which is an open-habitat, agricultural landscape specialist, did load strongly on this axis. The third trend was for certain large-gaped species to associate with large *Ficus* fruit sizes (in particular, the Green Imperial Pigeon, *Ducula aenea*, and Red-breasted Parakeet, *Psittacula alexandri*).

## Discussion

The limited scope of protected areas, combined with the extent of agricultural habitats across the world’s tropics, makes it critically important to assess the capacity of human-modified landscapes for biodiversity conservation [11]. We found that frugivorous birds interact with isolated *Ficus* trees independently of protected areas, although the scale of this interaction was highest at the forest edge. Overall, our results indicate that: 1) isolated *Ficus* trees are very important for avian frugivores in modified landscapes; 2) *Ficus* trees can conserve a large proportion of ecological function at such long distances that the avifaunas must survive independent of protected areas; 3) however, *Ficus* trees are no substitute for protected areas when it comes to conserving forest assemblages.

The importance of *Ficus* trees for forest frugivores is well established [15], [17]. However, the importance of isolated *Ficus* trees in modified habitats is little studied, and their potential role as micro-sites in matrix conservation is poorly understood [13], [20]. Here we found that they held richer frugivore assemblages, with higher abundance, than other isolated trees. The assemblages recorded in *Ficus* trees also differed in species composition from other isolated trees, indicating that they may support species that otherwise would not have adequate food resources in modified landscapes. The importance of isolated *Ficus* trees to frugivores is also indicated by the consistency of *Ficus*–frugivore interactions: 40% of species were recorded visiting more than half of the total trees surveyed. This is a similar figure to Luck and Daily’s [11] results from isolated *Miconia* trees in Costa Rica, where 43% visited more than half of the 40 trees surveyed. The number of individual birds recorded in many of the surveys was also impressive, with a high of 1,010 frugivores recorded landing in one tree during a three-hour survey, which may be the highest published number of birds recorded feeding in a single tree. These observations provide evidence that conserving isolated trees can support bird populations in modified landscapes, offering the chance to significantly improve seed dispersal and ecological connectivity [53]. Furthermore, our comparisons may provide useful guidance for practitioners and policy-makers setting priorities for matrix conservation, as isolated *Ficus* trees were more attractive to frugivores in this landscape than other tree types.

Further guidance can be gleaned from our regression results, which indicated that the characteristics of isolated *Ficus* trees had a major impact on the number of frugivores they held. All sites had similar compositions and numbers of habitat generalists (for example, *Pycnonotus cafer* was recorded at 119 of the 122 surveys, *Acridotheres tristis* at 118, and *Megalaima haemacephala* at 113, with mean abundances±standard error of 25.89±2.55, 19.57±1.70, and 11.38±1.35 respectively). However, larger trees, trees with lower surrounding land-use intensity, and trees closer to the forest, had higher frugivore abundances. Although *Ficus*-specific, the higher abundance of birds at trees with smaller fruit suggests that this fruit size may be easier to handle than larger fruit sizes. Both the boundary test and quantile regression indicated a significant relationship between the highest frugivore abundances and distance. In fact, the three trees with the highest abundance were all located within 1 km of the nearest forest, and eight of the 11 trees with more than 500 frugivores were located within 2 km. This suggests that local forests were able to support higher numbers of frugivores, which were attracted to these food resources, or that some flocking species were associated with forests, and were reluctant to visit *Ficus* trees at any considerable distance from the forest edge. Our incidence functions indicate that they were indeed reluctant to fly long distances from the forest, which supports the results of other studies on avian responses to landscape modification [6]. If conserving the composition of frugivores in a landscape is a conservation priority, the near complete absence of forest-dependent species at *Ficus* trees over 1 km from the forest indicates that isolated *Ficus* trees are no substitute for protected areas [54].

In a local context, the prominence of “season” in the GLM results is worth discussing. Isolated *Ficus* trees recorded higher species richness and abundance during the early and late monsoon compared to winter, with season accounting for a particularly high peak in frugivore abundances in the late monsoon. There are two possible explanations for this pattern: 1) fruit resources in protected areas may be seasonally scarce at particular times of year, and so frugivores venture further across human-modified landscapes to take advantage of isolated *Ficus* tree crops, which are available throughout the year; or 2) there is a large influx of migratory frugivores in the early- and especially the late- monsoon: but that these species are absent from the study area in winter. Although no local fruit availability research has been conducted, other tropical and subtropical studies report that fruit availability generally peaks during the monsoon season [16], [55], which makes the former hypothesis seem unlikely. Furthermore, at least one species, the Spot-winged Starling (*Saroglossa spiloptera*), is a known longitudinal migrant, while another, the Asian Koel (*Eudynamys scolopacea*), was conspicuously absent during the winter survey, despite being recorded in 79% of surveys during the monsoon months. We recorded particularly high numbers of Spot-winged Starlings during the late monsoon, with flock sizes reaching 110 birds in individual *Ficus* trees, and so expect that the presence of migratory species is responsible for the identification of season as an important predictor variable.

From a functional perspective, we also found declines in functional diversity with distance. Mean functional dispersion scores were 7.13% lower in trees over 25 km from the nearest forest compared to trees within 1 km. The abundance of frugivores with the largest gape widths also decreased markedly, suggesting that, along with declines in the number of seeds removed (as implied by the drop in the highest frugivore abundances), the range of seed sizes being dispersed may also fall with distance. However, although these results are concerning, functional diversity did not crash, as demonstrated by the most distant trees recording average scores of 92.87% of those on the forest edge. In this human-modified landscape at least, the majority of functional diversity can be conserved in the absence of local protected areas, and despite the loss of most forest-dependent species in matrix habitats.

The limited “sphere of influence” of protected areas in modified landscapes is reflected in our frugivore richness results. We found only a minor distance effect, which supports Eshiamwata et al.’s findings from isolated *Ficus* trees in Kenyan farmland, albeit at different spatial scales [13]. We feel the best explanation for the very small distance effect may lie in Sekercioglu et al.’s study of bird persistence in the Costa Rican agricultural landscape [56]. They found that birds reside in, rather than commute to, agricultural areas, making use in particular of isolated trees. In our study, the majority of species preferred and resided in modified habitats (as demonstrated by the incidence functions), and so richness would not be expected to decline with distance.

Close to the forest edge, another trend was apparent. Although there was no significant change in richness, there was some evidence of turnover in assemblage composition. Several forest dependent species were recorded at *Ficus* trees a few hundred metres from the forest, but were seemingly replaced by morphologically very similar species at greater distances. These include *Megalaima australis* replaced by *M*. *haemacephala*, *Pycnonotus flaviventris* replaced by *P*. *cafer*, and *Dendrocitta formosae* replaced by *Dendrocitta vagabunda*. The range of specific responses to distance, along with tree size, land-use intensity, and fruit size, was further illustrated in Fig 5. The inter-species variation provides support for Manning et al.’s continua-*Umwelt* view of variegated landscapes [57]. This approach recognizes the different responses of organisms to habitat disturbance, with species-specific environmental gradients and habitat preferences. In application to our dataset, it enables us to move beyond traditional forest specialist/matrix generalist categorizations [58], and identify the specific variables that individual species are responding to in modified landscapes. Furthermore, while we accept the notion of species-specific responses, grouping these species by their associations with particular variables also allows us to build conservation recommendations for targeted groups in modified landscapes.

In their recommendations for conserving seed dispersal functions in human-modified landscapes, McConkey et al. [21] suggest that functionally unique dispersers should be the focus of conservation efforts, which should aim to maintain their ecological function rather than just their minimum viable populations. In our study system, that would mean focusing conservation efforts on the hornbills, and in particular, the Great Indian Hornbill (*B*. *bicornis*). This would involve conserving large tracts of intact forest, as this species requires large foraging ranges [31], and only ventured further than 250 m into the matrix to feed at our isolated *Ficus* trees on one occasion. Nonetheless, in this scenario, the hornbill’s minimum viable population would be conserved, but its ecological function would be limited to those forested areas, and not improve the transfer of seeds across human-modified spaces. This species is not alone in its reluctance to cross human-modified habitats, as many large-bodied frugivores, which are often classified as functionally unique, are rare matrix visitors [7], [56]. Instead, the species recorded in isolated *Ficus* trees in our modified landscape were not clearly functionally unique, yet still supported a wide range of ecological function, even without the presence of the Great Indian Hornbill. While the loss of dispersal capacity for large seeded species may have undesirable ecological consequences [21], [53], basic avian seed dispersal appears to continue to function well across this network of isolated *Ficus* trees.

The *Ficus* trees in this study are dependent on the role avian frugivores play in dispersing seeds away from the parent tree, reducing mortality and increasing the chance of successful germination [14], [15]. As the protected areas in this landscape are believed to be too small to conserve low-density *Ficus* populations in the long term [22], the dispersal of *Ficus* seeds into modified habitats is crucial not only for their persistence, but also for the survival of their pollinator fig wasps (Agaonidae) [59]. If frugivores failed to provide effective seed dispersal services in this system, *Ficus* trees would in all likelihood be lost from the landscape, which would also cause the local co-extinction of the fig wasps, with a cascade effect on numerous other *Ficus* dependent arthropods [60]. The conservation of this relationship in disturbed landscapes should therefore be a priority in attempting to avert ecological collapse [14].

## Conclusions

Although human-modified landscapes are receiving greater attention in the literature, most studies focus on the Neotropics, and shade-coffee agrosystems in particular [6], [61]. Improving our understanding of functional group change in modified landscapes still represents a critical frontier in conservation science, and few studies have considered the matrix as a conduit for seed dispersal [6], [21]. Here, we present the results of the largest study undertaken on isolated trees, and one of the first to study *Ficu*s trees beyond the forests. We demonstrate that *Ficus* trees can be important tools in matrix conservation strategies, and may warrant preferential conservation ahead of other isolated trees. Given that *Ficus* trees are commonly found in many modified landscapes around the world’s tropics, our results may be applicable on a very broad geographical scale. Isolated *Ficus* trees can conserve frugivorous ecological function at such great distances from forests that the system is likely to be independent of protected areas, and *Ficus* trees hold more frugivorous species and individuals than other isolated trees. However, the quantity of seeds removed may decline with distance as the abundance of frugivores falls, and forest dependent species rarely venture more than a few hundred meters to feed in isolated *Ficus* trees. Therefore, while isolated *Ficus* trees may be among the best micro-sites for matrix conservation, they are still no substitute for protected areas in conserving forest dependent bird assemblages.

## Supporting Information

S1 Fig Incidence functions for all 33 frugivore species recorded in the study. Green curves represent forest dependent species, blue represent habitat generalists, and red represent matrix specialists. The curves are the predicted probability of occurrence, generated through a logistic regression model with distance from the nearest primary forest as the predictor variable for each species’ presence/absence data at 122 isolated *Ficus* trees. Species are a) Wedge-tailed Green Pigeon *Treron sphenura*; b) Orange-breasted Green Pigeon *Treron bicinctus*; c) Thick-billed Green Pigeon *Treron curvirostra*; d) Pin-tailed Green Pigeon *Treron apicauda*; e) Ashy-headed Green Pigeon *Treron phayrei*; f) Grey Treepie *Dendrocitta formosae*; g) Black-crested Bulbul *Pycnonotus flaviventris*; h) White-throated Bulbul *Alophoixus flaveolus*; i) Asian Fairy Bluebird *Irene puella*; j) Great Pied Hornbill *Buceros bicornis* k) Blue-eared Barbet *Megalaima australis*; l) Oriental Pied Hornbill *Anthracoceros albirostris*; m) Spot-winged Starling *Saroglossa spiloptera*; n) Hill Myna *Gracula religiosa*; o) Eastern Jungle Crow *Corvus levaillantii*; p) Green Imperial Pigeon *Ducula aenea*; q) Chestnut-tailed Starling *Sturnus malabarica*; r) Jungle Myna *Acridotheres fuscus*; s) Asian Pied Starling *Gracupica contra*; t) Rufous Treepie *Dendrocitta vagabunda*; u) Red-vented Bulbul *Pycnonotus cafer*; v) Coppersmith Barbet Megalaima haemacephala; w) Blue-throated Barbet *Megalaima asiatica*; x) Red-whiskered Bulbul *Pycnonotus jocosus*; y) Common Myna *Acridotheres tristis*; z) Yellow-footed Green Pigeon *Treron phoenicopterus*; aa) Asian Koel *Eudynamys scolopacea*; ab) Lineated Barbet *Megalaima lineata*; ac) Great Myna *Acridotheres grandis*; ad) Black-hooded Oriole *Oriolus xanthornus*; ae) Golden-fronted Leaf-bird *Chloropsis aurifrons*; af) Rose-ringed Parakeet *Psittacula krameri*; ag) Red-breasted Parakeet *Psittacula alexandri*. The figures were constructed using the R package “GGplot2” (Wickham, 2009).
(TIFF)

S1 Table Differences between species richness, abundance, and functional diversity parameters across the three tree categories, controlled by canopy area. Values are means ± standard error. Different superscript letters denote significantly different means. Abundance is the mean number of individual frugivores recorded in each tree per m^2^, and presented in tree categories. Observed richness is the mean number of frugivorous bird species recorded in the surveys of each tree per m^2^, presented by category. FDis is a multidimensional index of the mean distance of an individual species to the centroid of all species in the community (Laliberté & Legendre, 2010). Different superscript letters denote significantly different means at *p*<0.05 using ANOVA with Welch’s F and Games–Howell post hoc tests. Although this test found that isolated *Ficus* trees still have higher frugivore abundances than the other two tree types when area is controlled, there was no significant difference in richness between *Ficus* and isolated fruit trees, while isolated fruit trees had higher FDis/m^2^ of canopy area. These results may be explained by the exceptionally large canopy areas that were used to divide *Ficus* richness and FDis scores, which cannot vary as widely as abundance records (as, in the case of richness, there was only a maximum of 33 frugivores in the study). This result verifies the importance of tree size for frugivore abundance, species richness, and FDis. We elected to exclude area-controlled results from the main analysis as we were more interested in using trees as the unit of study, as this can more directly be influenced by conservation measures. Furthermore, we felt that having a large canopy area was intrinsic to the advantage *Ficus* trees may possess over other species in terms of frugivore conservation, and so controlling for area would constrain our ability to compare the actual conservation value of each tree type.
(DOCX)

S1 Appendix Differences between species richness, abundance, and functional diversity parameters across the three tree categories, controlled by canopy area. To investigate the effect of area on frugivore richness, abundance, and FDis values in isolated *Ficus* trees, we conducted an additional analysis of these three properties across *Ficus*, fruit, and large trees when controlling for area. We used canopy area as the best proxy for area, and divided richness, abundance, and FDis by canopy area for each tree. After examining the assumptions of normality and heteroscedasticity, the new average values for each tree category were compared using ANOVA with Welch’s F and Games–Howell post hoc tests. Means and differences at the *p*<0.05 level of significance are presented in S1 Table.
(DOCX)

S2 Appendix Distance measurement. To test the adequacy of fit between either distance from isolated *Ficus* trees to the nearest protected area, or to the nearest forest of any quality and frugivore abundance, we conducted a linear regression. We ran a linear regression model for each distance measure in turn, and found that the distance from protected area had a slightly better fit (*R* ^2^ = 0.19) than the distance from the nearest forest of any quality (*R* ^2^ = 0.18). Although the difference was only marginal, we therefore decided to use distance from the nearest protected area as our distance measure throughout the analyses.
(DOCX)
