# Supplementary figures and images for: Are Protected Areas Required to Maintain Functional Diversity in Human-Modified Landscapes?
Source: PLoS One. 2015 May 6;10(5):e0123952. doi: 10.1371/journal.pone.0123952 (PMC4422652; doi:10.1371/journal.pone.0123952)

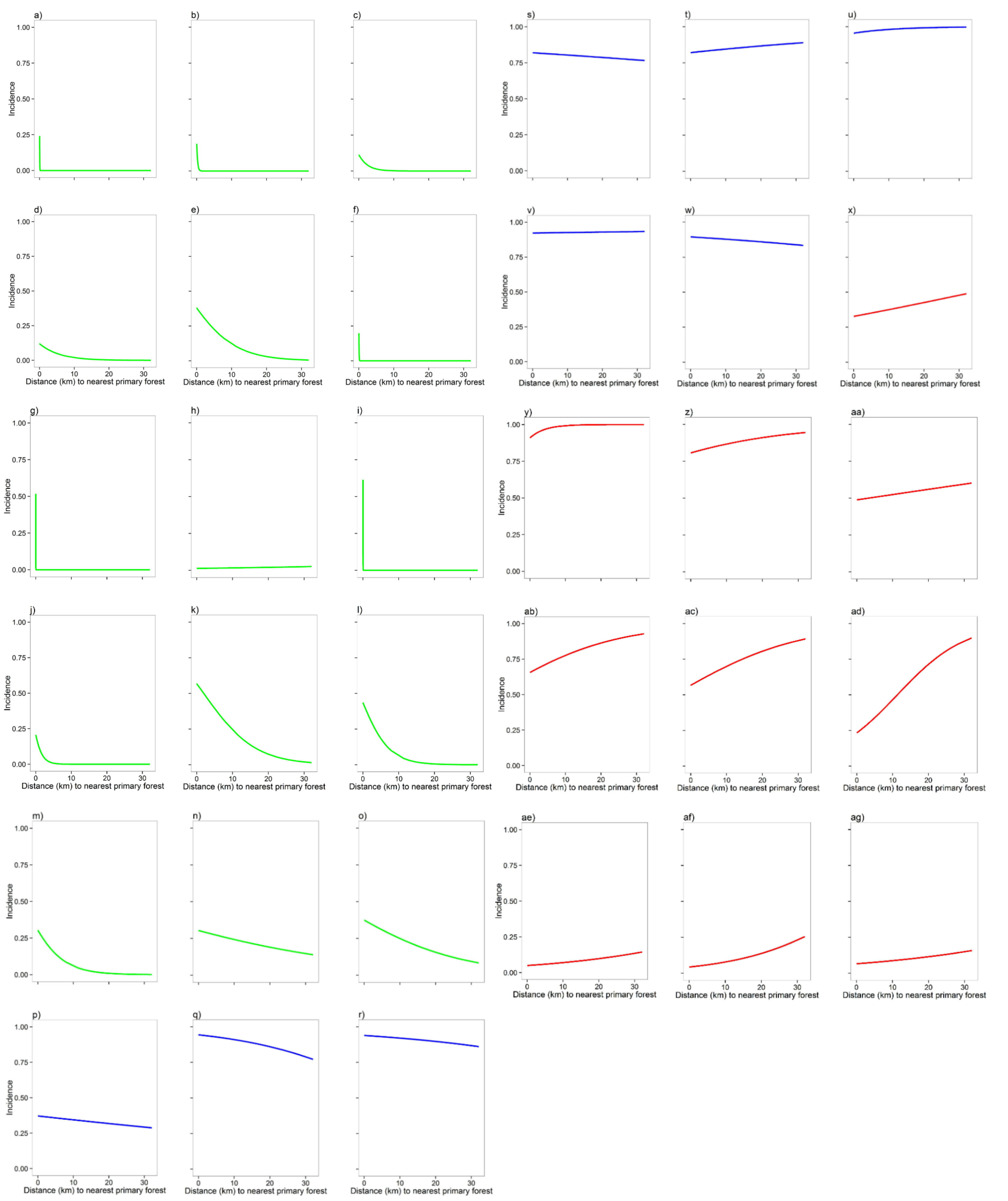

Supplement: S1 Fig — Green curves represent forest dependent species, blue represent habitat generalists, and red represent matrix specialists. The curves are the predicted probability of occurrence, generated through a logistic regression model with distance from the nearest primary forest as the predictor variable for each species’ presence/absence data at 122 isolated Ficus trees. Species are a) Wedge-tailed Green Pigeon Treron sphenura; b) Orange-breasted Green Pigeon Treron bicinctus; c) Thick-billed Green Pigeon Treron curvirostra; d) Pin-tailed Green Pigeon Treron apicauda; e) Ashy-headed Green Pigeon Treron phayrei; f) Grey Treepie Dendrocitta formosae; g) Black-crested Bulbul Pycnonotus flaviventris; h) White-throated Bulbul Alophoixus flaveolus; i) Asian Fairy Bluebird Irene puella; j) Great Pied Hornbill Buceros bicornis k) Blue-eared Barbet Megalaima australis; l) Oriental Pied Hornbill Anthracoceros albirostris; m) Spot-winged Starling Saroglossa spiloptera; n) Hill Myna Gracula religiosa; o) Eastern Jungle Crow Corvus levaillantii; p) Green Imperial Pigeon Ducula aenea; q) Chestnut-tailed Starling Sturnus malabarica; r) Jungle Myna Acridotheres fuscus; s) Asian Pied Starling Gracupica contra; t) Rufous Treepie Dendrocitta vagabunda; u) Red-vented Bulbul Pycnonotus cafer; v) Coppersmith Barbet Megalaima haemacephala; w) Blue-throated Barbet Megalaima asiatica; x) Red-whiskered Bulbul Pycnonotus jocosus; y) Common Myna Acridotheres tristis; z) Yellow-footed Green Pigeon Treron phoenicopterus; aa) Asian Koel Eudynamys scolopacea; ab) Lineated Barbet Megalaima lineata; ac) Great Myna Acridotheres grandis; ad) Black-hooded Oriole Oriolus xanthornus; ae) Golden-fronted Leaf-bird Chloropsis aurifrons; af) Rose-ringed Parakeet Psittacula krameri; ag) Red-breasted Parakeet Psittacula alexandri. The figures were constructed using the R package “GGplot2” (Wickham, 2009). (TIFF) [file pone.0123952.s001.tiff]
