# Supplementary material for: Are Protected Areas Required to Maintain Functional Diversity in Human-Modified Landscapes?
Source: PLoS One. 2015 May 6;10(5):e0123952. doi: 10.1371/journal.pone.0123952 (PMC4422652; doi:10.1371/journal.pone.0123952)
Supplement: S1 Table — Values are means ± standard error. Different superscript letters denote significantly different means. Abundance is the mean number of individual frugivores recorded in each tree per m2, and presented in tree categories. Observed richness is the mean number of frugivorous bird species recorded in the surveys of each tree per m2, presented by category. FDis is a multidimensional index of the mean distance of an individual species to the centroid of all species in the community (Laliberté & Legendre, 2010). Different superscript letters denote significantly different means at p<0.05 using ANOVA with Welch’s F and Games–Howell post hoc tests. Although this test found that isolated Ficus trees still have higher frugivore abundances than the other two tree types when area is controlled, there was no significant difference in richness between Ficus and isolated fruit trees, while isolated fruit trees had higher FDis/m2 of canopy area. These results may be explained by the exceptionally large canopy areas that were used to divide Ficus richness and FDis scores, which cannot vary as widely as abundance records (as, in the case of richness, there was only a maximum of 33 frugivores in the study). This result verifies the importance of tree size for frugivore abundance, species richness, and FDis. We elected to exclude area-controlled results from the main analysis as we were more interested in using trees as the unit of study, as this can more directly be influenced by conservation measures. Furthermore, we felt that having a large canopy area was intrinsic to the advantage Ficus trees may possess over other species in terms of frugivore conservation, and so controlling for area would constrain our ability to compare the actual conservation value of each tree type. (DOCX) [file pone.0123952.s002.docx]

| **Parameter** | ***Ficus*** | **Fruit** | **Large** |
| --- | --- | --- | --- |
| Abundance | 0.64±0.05 ^a^ | 0.20±0.04 ^b^ | 0.08±0.03 ^c^ |
| Observed richness | 0.04±0.002 ^a^ | 0.06±0.01 ^a^ | 0.03±0.01 ^b^ |
| FDis | 0.003±0.0001 ^a^ | 0.01±0.003 ^b^ | 0.006±0.001 ^a,c^ |

**S1 Table:** **Differences between species richness, abundance, and functional diversity parameters across the three tree categories, controlled by canopy area.**
