# Supplementary material for: Are Protected Areas Required to Maintain Functional Diversity in Human-Modified Landscapes?
Source: PLoS One. 2015 May 6;10(5):e0123952. doi: 10.1371/journal.pone.0123952 (PMC4422652; doi:10.1371/journal.pone.0123952)
Supplement: S1 Appendix — To investigate the effect of area on frugivore richness, abundance, and FDis values in isolated Ficus trees, we conducted an additional analysis of these three properties across Ficus, fruit, and large trees when controlling for area. We used canopy area as the best proxy for area, and divided richness, abundance, and FDis by canopy area for each tree. After examining the assumptions of normality and heteroscedasticity, the new average values for each tree category were compared using ANOVA with Welch’s F and Games–Howell post hoc tests. Means and differences at the p<0.05 level of significance are presented in S1 Table. (DOCX) [file pone.0123952.s003.docx]

**S1 Appendix. Differences between species richness, abundance, and functional diversity parameters across the three tree categories, controlled by canopy area.**

To investigate the effect of area on frugivore richness, abundance, and FDis values in isolated *Ficus* trees, we conducted an additional analysis of these three properties across *Ficus*, fruit, and large trees when controlling for area. We used canopy area as the best proxy for area, and divided richness, abundance, and FDis by canopy area for each tree. After examining the assumptions of normality and heteroscedasticity, the new average values for each tree category were compared using ANOVA with Welch’s F and Games–Howell post hoc tests. Means and differences at the *p*<0.05 level of significance are presented in S1 Table.

**S1 Table:** Differences between species richness, abundance, and functional diversity parameters across the three tree categories, controlled by canopy area.

| **Parameter** | ***Ficus*** | **Fruit** | **Large** |
| --- | --- | --- | --- |
| Abundance | 0.64±0.05 ^a^ | 0.20±0.04 ^b^ | 0.08±0.03 ^c^ |
| Observed richness | 0.04±0.002 ^a^ | 0.06±0.01 ^a^ | 0.03±0.01 ^b^ |
| FDis | 0.003±0.0001 ^a^ | 0.01±0.003 ^b^ | 0.006±0.001 ^a,c^ |

Values are means ± standard error. Different superscript letters denote significantly different means. Abundance is the mean number of individual frugivores recorded in each tree per m^2^, and presented in tree categories. Observed richness is the mean number of frugivorous bird species recorded in the surveys of each tree per m^2^, presented by category. FDis is a multidimensional index of the mean distance of an individual species to the centroid of all species in the community [38]. Different superscript letters denote significantly different means at *p*<0.05 using ANOVA with Welch’s F and Games–Howell post hoc tests.

Although this test found that isolated *Ficus* trees still have higher frugivore abundances than the other two tree types when area is controlled, there was no significant difference in richness between *Ficus* and isolated fruit trees, while isolated fruit trees had higher FDis/m^2^ of canopy area. These results may be explained by the exceptionally large canopy areas that were used to divide *Ficus* richness and FDis scores, which cannot vary as widely as abundance records (as, in the case of richness, there was only a maximum of 33 frugivores in the study). This result verifies the importance of tree size for frugivore abundance, species richness, and FDis. We elected to exclude area-controlled results from the main analysis as we were more interested in using trees as the unit of study, as this can more directly be influenced by conservation measures. Furthermore, we felt that having a large canopy area was intrinsic to the advantage *Ficus* trees may possess over other species in terms of frugivore conservation, and so controlling for area would constrain our ability to compare the actual conservation value of each tree type.
