# Supplementary material for: Are Protected Areas Required to Maintain Functional Diversity in Human-Modified Landscapes?
Source: PLoS One. 2015 May 6;10(5):e0123952. doi: 10.1371/journal.pone.0123952 (PMC4422652; doi:10.1371/journal.pone.0123952)
Supplement: S2 Appendix — To test the adequacy of fit between either distance from isolated Ficus trees to the nearest protected area, or to the nearest forest of any quality and frugivore abundance, we conducted a linear regression. We ran a linear regression model for each distance measure in turn, and found that the distance from protected area had a slightly better fit (R 2 = 0.19) than the distance from the nearest forest of any quality (R 2 = 0.18). Although the difference was only marginal, we therefore decided to use distance from the nearest protected area as our distance measure throughout the analyses. (DOCX) [file pone.0123952.s004.docx]

**S2 Appendix. Distance measurement.**

To test the adequacy of fit between either distance from isolated *Ficus* trees to the nearest protected area, or to the nearest forest of any quality and frugivore abundance, we conducted a linear regression. We ran a linear regression model for each distance measure in turn, and found that the distance from protected area had a slightly better fit (*R*^2^=0.19) than the distance from the nearest forest of any quality (*R*^2^=0.18). Although the difference was only marginal, we therefore decided to use distance from the nearest protected area as our distance measure throughout the analyses.
